# Assessment of the Diaphragm Thickness Decrease in Critically Ill COVID-19 Patients: Could Computed Tomography Be of Aid Regarding Diaphragm Muscle Mass?

**DOI:** 10.7759/cureus.47195

**Published:** 2023-10-17

**Authors:** Oana-Elena Branea, Sanda Maria Copotoiu, Diana Andreea Becica, AnaMaria Romina Budeanu, Razvan Gabriel Budeanu, Mihai Emanuel Becica, Dragos Constantin Cucoranu, Septimiu Voidazan, Monica Chis, Alexandra Elena Lazar

**Affiliations:** 1 Discipline of Anesthesia and Intensive Care, George Emil Palade University of Medicine, Pharmacy, Science and Technology, Târgu Mureș, ROU; 2 Department of Anesthesia and Intensive Care, Târgu Mureș County Emergency Clinical Hospital, Târgu Mureș, ROU; 3 Department of Radiology, Târgu Mureș County Emergency Clinical Hospital, Târgu Mureș, ROU; 4 Department of Radiology, Târgu Mureș County Emergency Clinical Hospital, Targu Mures, ROU; 5 Discipline of Epidemiology, George Emil Palade University of Medicine, Pharmacy, Science and Technology, Târgu Mureș, ROU; 6 Department of Epidemiology, Târgu Mureș County Emergency Clinical Hospital, Târgu Mureș, ROU; 7 Discipline of Rheumatology, George Emil Palade University of Medicine, Pharmacy, Science and Technology, Târgu Mureș, ROU; 8 Department of Rheumatology, Târgu Mureș County Emergency Clinical Hospital, Târgu Mureș, ROU

**Keywords:** muscle alteration, diaphragm thickness, computed tomography, covid-19, critical care

## Abstract

Introduction: The diaphragm has a significant clinical value on respiratory performance. There is little literature on the use of thorax computed tomography for the purpose of identifying alterations in diaphragm thickness in critically ill patients diagnosed with COVID-19. The present study aims to investigate dynamic changes in muscle thickness and its association with clinical outcomes.

Methods: A single-center retrospective observational study was conducted in a tertiary intensive care unit (ICU). The study comprised adult patients with severe COVID-19 who were admitted to the ICU and underwent two thorax CT scans. We measured diaphragmatic thickness at the level of the celiac truncus.

Results: The average reduction in thickness of the dynamic diaphragm was found to be -0.58 mm for the right diaphragm and -0.54 mm for the left diaphragm. The diaphragm thickness exhibited a substantial decrease on both the right and left sides in both CT scans (p=0.02). A negative correlation coefficient was observed for both the right and left diaphragm. The criterion indicating a poor prognosis for the right diaphragm was a value greater than -0.175, whereas it was more significant for the left diaphragm than -0.435. The cut-off values indicated a high risk of prolonged mechanical ventilation and an increased risk of ICU mortality.

Conclusion: CT diaphragm evaluation in mechanically ventilated COVID-19 patients has the possibility of becoming a reliable tool for predicting muscle modifications.

## Introduction

Unknown to humanity until recently, the coronavirus pandemic of 2019 (COVID-19) resulted in serious and critical consequences worldwide [1,2]. The COVID-19 pandemic occurred because of the severe acute respiratory syndrome coronavirus 2 (SARS-CoV-2), and, since its introduction in December 2019, it has caused more than six million deaths worldwide [3]. In response to COVID-19’s global spread and severity, the World Health Organization (WHO) declared COVID-19 a public health emergency on January 30, 2020, and a pandemic on March 11, 2020 [4,5]. Patients diagnosed with COVID-19 were classified from mild to severe [6], and critical cases were characterized by acute respiratory failure, shock, multiple organ dysfunction, and high mortality rates [7]. As such, thorax computed tomography (CT) is one of the most practical and rapid evaluation approaches that can aid critically ill COVID-19 patients, and it is widely accepted for diagnosing the severity and prognosis of COVID-19 [8,9]. Although CT is regarded as the gold standard for determining total skeletal muscle quantity [10], nowadays it is viewed as a possible means to predict disease severity and patient prognosis [11,12]. Quantitative tissue measures can be acquired using CT in a very reproducible manner, and some data derived from CT have been shown to strongly correlate with clinical outcomes [11,12].

Because at least a chest CT examination is recommended for COVID-19 diagnosis, diaphragm thickness measurement can be analyzed by the mediastinal window of the chest CT image [12]. Identifying accurate measurements of crus thickness is made easier by using the L1 vertebral body and the celiac artery as landmarks. A further benefit of CT is it displays the diaphragm in 3D multiplanar reconstruction though it does not illustrate diaphragmatic mobility. There are several potential challenges that could intricate CT scan measurements of the diaphragm thickness; for example: demographic differences in age and gender, as well as variations in spontaneous or assisted respiration [13]. The most essential inhalation muscle is the diaphragm, which contributes more than 60%-80% of the force needed to inhale [14]. Muscle atrophy is a ubiquitous and serious issue among patients in ICUs. As a result, the investigation of diaphragm atrophy degree is particularly notable in the context of patients who are mechanically ventilated. In comparison to psoas major and limb muscle atrophy, the diaphragm may have a more direct effect on respiratory performance and prognosis [12,14]. The diaphragm may therefore have a greater clinical value as a nutritional evaluation indicator. Early and persistently elevated catabolism and the associated atrophy of skeletal muscle will impact the diaphragm in critical illness [11,15]. The diaphragm will also expand similarly to the psoas major and limb muscles once the main condition has been treated and anabolism increases, reflecting the patient’s nutritional state [15]. Diaphragm atrophy can occur in critically ill individuals undergoing mechanical ventilation (MV) for a variety of causes. In animal models, adult rats exposed to MV for 6 hr showed significantly reduced protein synthesis [16]. Diaphragm biopsies also showed diminished cross-sectional muscle fiber areas in humans exposed to more than 18 hr of MV [17]. The prognostic value of diaphragm thickness measured using thoracic CT images, which have been widely used for COVID-19 patient diagnosis, stratification, and monitoring during the COVID-19 pandemic, was only assessed in a few studies [11,12]. For example, You et al. identified diaphragm thickness as a nutritional assessment and hospital stay prediction [11], along with Parlak et al., who demonstrated a low diaphragm thickness and density were associated with severe disease in patients with COVID-19 and could be evaluated as poor prognostic markers [12]. Considering these aspects as a motivation for the present study, the main hypothesis was that diaphragm muscles in critically ill COVID-19 patients decreased in thickness during hospitalization with MV and that diaphragm alteration is associated with poor clinical outcomes. 

## Materials and methods

Study design

This single-center retrospective observational cohort study was conducted in the Intensive Care Unit (ICU) of Târgu Mureș County Emergency Clinical Hospital, Romania. The study was approved by the Institutional Ethics Committee (12964/20.05.2021 and 2616/10.02.2023), and it was piloted in compliance with the Declaration of Helsinki. As the data collected for this study were anonymous, the requirement for informed consent was waived for all enrolled patients. 

Study population and data collection

**Figure 1:**
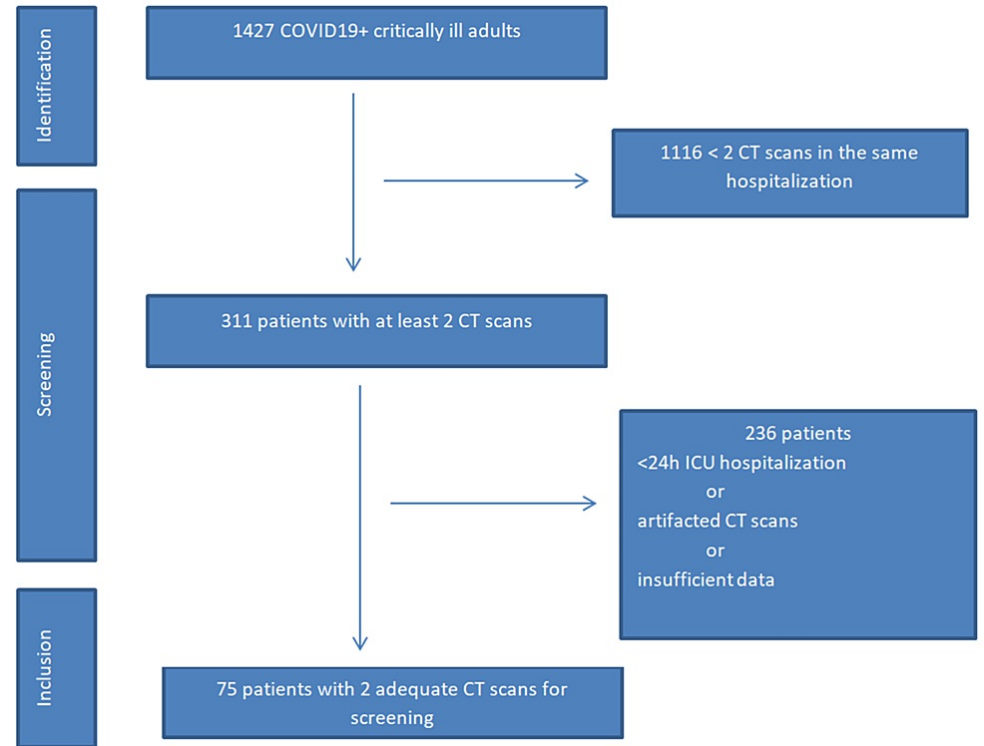
COVID-19 critically ill patients' flowchart

This study identified all critically ill positive COVID-19 patients admitted to the ICU from August 2020 to December 2022. Patients included in the study were adults admitted to the ICU for at least 24 hr who required any type of oxygen therapy and had at least two CT examinations during their ICU stay (at more than 24 hr apart). Exclusion criteria included noncritical ill patients, or critically ill ones who tested negative for COVID-19 reverse transcriptase polymerase chain reaction, who were under the age of 18 years, who received critical care support for less than 24 hr, and for whom less than two diaphragm CT scan investigations were performed during the same hospitalization. All CT scans were screened for adequacy. Nondiagnostic CT scans (due to incomplete field of view, artifacts, or patient movements) and CT scan investigations completed in less than 24 hr were excluded. Figure 1 shows the study population inclusion and exclusion flowchart.

Furthermore, data on demographics, comorbidities (Charlson Comorbidity Index that predicts 10-year survival in patients with multiple comorbidities [18]), ICU admission status, and diagnosis, clinical follow-up of the respiratory disease progression (need for noninvasive ventilation (NIV) or invasive MV (IMV)), along with the time period between CT scans, length of stay in ICU and hospital, and mortality, were collected from the electronic medical records (Hipocrate3 Concept). In addition, laboratory results were identified for each patient. The set of blood tests included inflammatory markers: white blood cell (WBC) count, absolute neutrophil count, absolute lymphocyte count, platelets count, C-reactive protein (CRP), interleukin-6 (IL-6), and metabolic markers such as albumin, glucose, creatinine, glomerular filtration rate (GFR), fibrinogen, and ferritin. The neutrophil-to-lymphocyte ratio (NLR) was calculated by dividing the absolute neutrophil count by the absolute lymphocyte count. The platelet-to-lymphocyte ratio was calculated by dividing the absolute platelet count by the absolute lymphocyte count. The systemic inflammation index was defined as neutrophil count monocyte/lymphocyte count. The Quick COVID-19 Severity Index (qCSI) was used to predict 24 hr risk of critical respiratory illness and scores ≥7 points classify patients (ns2) at high-intermediate and high risk [19]. The COVID Home Safely Now (CHOSEN) Risk Score was used to predict suitability for discharge and scores <19 points (for patients without albumin available) and <29 points (for patients with albumin available) assess patients as being unlikely suitable for discharge [20]. This study population was divided based on the outcome (survivors and deceased), respectively, on the decrease in diaphragm thickness (with or without alteration).

Diaphragm analysis CT protocol

Computed Tomography

Patients with two successive examinations were included in the study group to observe the musculoskeletal changes. CT images were obtained using 64-slice CT machines with 120 kV and automatic mA. The inspiratory breath-hold command was used only in nonsedated patients. Axial images were reconstructed using a slice thickness of 1 mm and a standard b31f kernel. Images were obtained from the hospital’s Picture Archiving and Communicating System, (ns1) and the RadiAnt DICOM Viewer was used in data analysis.

Image Assessment

**Figure 2:**
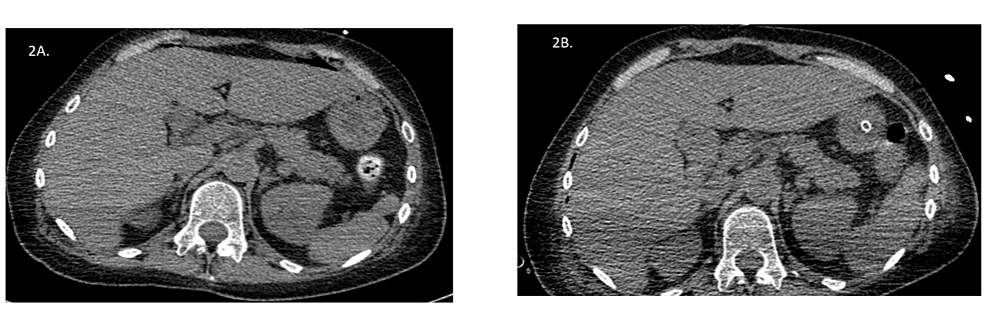
Illustrative images of the decrease in diaphragm thickness assessed by CT scan in two different time points of patient’s hospitalization (A) Diaphragm thickness assessment in the first CT investigation (right diaphragm thickness 5.78 mm and left diaphragm thickness 4.89 mm). (B) Diaphragm thickness assessment in the second CT investigation (right diaphragm thickness 5.01 mm and left diaphragm thickness 4.77 mm).

The images were assessed by two radiologists (2+ years of experience). Radiologists were blind to patients’ evolution and outcome. CT images were evaluated in the abdominal window (window center, 60 HU; window width, 400 HU), and magnification was freely modifiable. Diaphragmatic thickness was measured at the level of celiac truncus, emerging in a plane that crosses the anterior vertebral body border, as was previously demonstrated in the literature [13,21]. The image assessment is presented in Figures 2A, 2B.

Data processing and statistical analysis

All data were organized in Excel sheets and statistically analyzed. Statistical analysis was performed using SPSS, version 27 (IBM Corp., Armonk, NY, USA). Continuous data are presented as medians (minimum-maximum) or means (±standard deviation), with categorical data as proportions. The Kolmogorov-Smirnov test was used to assess the normal distribution of the continuous numerical variables. Statistical differences between groups were assessed with the chi-square test using Yates’s correction or Fisher’s exact test, when appropriate, for categorical variables. The Mann-Whitney U test was used for non-Gaussian variables, and a Student’s t-test was used for the Gaussian continuous variables. Intervariability was performed by the Bland and Altman methods. Receiver-operating characteristics (ROC) curve analysis was performed for biomarkers and combinations. Cut-off values regarding diaphragm thickness and MV period in patient outcome were determined using Youden’s Index. Area under the curve (AUC) values were displayed including a 95% confidence interval (CI) and compared according to Hanley and McNeil’s method. For correlations, Spearman’s coefficients were used. Univariate and multivariate logistic regression analyses were performed for biomarkers, and odds ratios (ORs) were displayed. A p-value of less than 0.05 was considered statistically significant.

## Results

Characteristics of the 75 patients included in the study

Table 1 summarizes the baseline characteristics of included patients. The 75-patient study population represented 5.25% of the total 1,427 COVID-19 admissions in the tertiary ICU over the study period (August 1, 2020 to December 31, 2022; Figure 1).

**Table 1:** Critically ill patients’ characteristics and ICU outcome for survivors and deceased groups ICU = Intensive Care Unit; ARF = acute respiratory failure; CCI = Charlson Comorbidity Index; CT = computed tomography; qCSI = Quick COVID-19 Severity Index; CHOSEN = COVID-19 Home Safely Now Risk Score; LOS = length of stay; CTscan1 = first CT scan investigation; CTscan2 = second CT scan investigation; * = Chi-Square test; ** = Student t-test; *** = Mann-Whitney U test.

	All patients (n= 75)	Deceased (n = 56)	Survivors (n = 19)	P
Demographics				
Male sex (n, %)	40 (53.3)	27 (48.2)	13 (68.4)	0.18*
Age (years, mean+/-SD)	67.9+/-14.3	69.66+/-14.6	62.68+/-12.6	0.67**
ICU Admission Diagnostics				
ARF (n, %)	58 (77.33)	43 (76.8)	15 (78.9)	0.65*
Other (n, %)	17 (22.6)	13 (23.2)	4 (21.1)	0.65*
Comorbidities				
Respiratory disease	23 (30.7)	18 (32.1)	5 (26.3)	0.77*
Cardiovascular disease	68 (90.7)	51 (91.1)	17 (89.5)	0.83*
Diabetes	26 (34.7)	20 (35.7)	6 (31.6)	0.78*
Obesity	16 (21.3)	11 (19.6)	5 (26.3)	0.74*
Neurological disease	27 (36.0)	21 (37.5)	6 (31.6)	0.78*
Dementia	15 (20.0)	11 (19.6)	4 (21.1)	0.89*
Chronic kidney disease	16 (21.3)	15 (26.8)	1 (5.3)	0.056*
Hepatic disease	21 (28.0)	14 (25.0)	7 (36.8)	0.38*
Anemia	43 (57.3)	35 (62.5)	8 (42.1)	0.18*
Malignity	8 (10.7)	6 (10.7)	2 (10.5)	0.98*
CCI (median, min-max)	7 (0-13)	8 (0-13)	6 (2-12)	0.062***
Mild 1–2 (n, %)	3 (4)	1 (1.8)	2 (10.6)	0.08*
Moderate 3–4 (n, %)	7 (8.3)	5 (9.0)	2(10.6)	0.26*
Severe ≥5 (n, %)	65 (86.6)	50 (89.3)	15 (78.9)	0.56*
qCSI				
≤6 (n, %)	7 (9.33)	4 (7.14)	3 (15.8)	0.50*
≥7 (n, %)	68 (90.6)	52 (92.85)	16 (84.2)	0.50*
CHOSEN Score				
<29 for patients with albumin available (n, %)	58 (77.3)	42 (75.0)	16 (84.2)	0.61*
<19 for patients with albumin not available (n, %)	7 (9.33)	6 (10.7)	1 (5.55)	0.83*
LOS Hospital (days, median, min-max)	18 (2-91)	15.5 (2-91)	23 (14-73)	0.002***
LOS ICU (days, median, min-max )	12 (2-57)	12 (2-91)	14 (3-57)	0.50***
Time Period Between CT1 and CT2 (days, median, min-max )	9 (1-56)	7 (1-28)	12 (2-56)	0.004*

The majority were male (53.3%), sexagenarian (67.9+/-14.3 years of age), and admitted for acute respiratory failure (ARF; 77.33%). All patients had an emergency criterion to be admitted to the ICU. There was no statistically significant difference regarding sex (p = 0.18), age (p = 0.67), and ICU admission diagnosis (p = 0.65) between survivors and deceased patients. Patients had a variety of comorbidities, including cardiovascular diseases (90.7%), anemia (57.3%), neurological disease (36.0%), diabetes (34.7%), and respiratory disease (30.7%). No statistically significant differences were determined in assessing comorbidities when comparing survivors and deceased (p > 0.05). The same remark was made regarding comorbidities score, whereas more than 85% had a severe Charlson Comorbidity Index (CCI ≥ 5 points). Used as a prognostic tool for early clinical decompensation, the qCSI identified 90.6% of patients at risk of critical respiratory illness in the next 24 hr (qCSI ≥ 7 points). At the same time, the CHOSEN score, a predictor of suitability for discharge, identified more than 85% of patients not apt to be discharged (58 patients presented with a CHOSEN score of <29 points and seven patients presented a CHOSEN score of <19 points). The LOS in the ICU was 12 days (min-max 2-57), with no difference between survivors and deceased (p = 0.50), but survivors had a significantly longer LOS in the hospital compared to the deceased (p = 0.002). The period between the first CT scan performed (CT1) and the second CT scan performed (CT2) was nine days (min-max 1-56). The total ICU mortality rate for the cohort was 85.75%.

Status upon ICU admission and respiratory support during ICU stay

Table 2 analyzes critically ill patients’ admission status.

**Table 2:** Patients’ status upon ICU admission and respiratory support during ICU stay ICU = Intensive Care Unit; GCS = Glasgow Coma Scale (points); MAP = mean arterial pressure (mmHg); SpO_2_ = peripheral oxygen saturation (%); NIV-CPAP = noninvasive ventilation continuous positive airway pressure; IMV = invasive mechanical ventilation; MV = mechanical ventilation; * = Chi-Square test, ** = Student t-test.

	All patients (n = 75)	Deceased (n = 56)	Survivors (n = 19)	P
ICU Admission Status				
Neurological				
GCS > 8 (n, %)	68 (91.8)	50 (85.7)	18 (94.1)	0.57*
GCS ≤ 8 (n, %)	7 (8.2)	6 (14.3)	1 (5.9)	0.57*
Hemodynamic				
MAP (mean+/-SD)	89.1+/-18.87	88.03+/-17.9	92.02+/-21.5	0.42**
Respiratory				
SpO_2_ (mean+/-SD)	89.7+/-10.1	89.89+/-10.1	89.1+/-10.3	0.78**
SpO_2_ ≤ 92% (n, %)	39 (52.0)	29 (51.78)	10 (52.6)	0.65*
ICU Admission Oxygenation				
Facial mask (n, %)	35 (46.7)	21 (37.5)	14 (73.7)	0.008*
NIV-CPAP (n, %)	17 (22.7)	14 (25.0)	3 (15.8)	0.40*
IMV (n, %)	23 (30.7)	21 (37.5)	2 (10.5)	0.042*
ICU Stay Oxygenation				
No need for NIV or IMV (n, %)	13 (17.3)	0 (0.0)	13 (68.4)	0.0001*
Need for NIV-CPAP (n, %)	32 (42.7)	21 (37.5)	11 (57.9)	0.18*
Need for IMV (n, %)	62 (82.7)	56 (100.0)	6 (31.6)	0.0001*
IMV ≤24 hr (n, %)	12 (16.0)	12 (21.4)	0 (0.0)	0.030*
IMV 25 hr–96 hr (n, %)	10 (13.3)	8 (14.3)	2 (10.5)	0.74*
IMV >96 hr (n, %)	40 (53.3)	36 (64.3)	4 (21.1)	0.001*
Successful MV weaning (n, %)	8 (10.7)	3 (5.4)	5 (26.3)	0.022*
Unsuccessful MV weaning (n, %)	4 (5.3)	3 (5.4)	1 (5.3)	0.98*
Tracheostomy (n, %)	3 (4.0)	3 (5.4)	0 (0.0)	0.56*

No statistically significant differences between survivors and deceased groups were presented when evaluating neurological, hemodynamic, and respiratory status (p > 0.05). The results showed that 8.2% of all patients had severe neurologic impairment (GCS ≤ 8 points), mean arterial pressure (MAP) was over 65 mmHg for the entire cohort, and 52% of patients expressed respiratory signs of hypoxia as SpO2 dropped below 92%, with a mean of 89.7+/-10.1%. When admitted to the ICU, 46.7% received facial mask oxygenation. Respiratory management consisted of NIV-CPAP for 42.7% of patients and further IMV for 82.7%, showing a statistically significant difference between survivors and deceased patients (p = 0.0001). A statistically significant difference between survivors and deceased patients in weaning from MV was demonstrated between the two groups (p = 0.022).

Diaphragm thickness assessment using CT scan

Evaluation of the diaphragm thickness was assessed by two independent radiologists with more than two years of experience. Table 3 illustrates the differences in their evaluations.

**Table 3:** Diaphragm thickness in first and second CT scan investigation CT1 = first CT scan investigation; CT2 = second CT scan investigation; A = first radiologist investigator results; B = second radiologist investigator results; A+B = mean results of investigators A and B.

	Mean right diaphragm thickness investigator A (mm)	Mean right diaphragm thickness investigator B (mm)	Mean left diaphragm thickness investigator A (mm)	Mean left diaphragm thickness investigator B (mm)	Mean right diaphragm thickness A+B (mm)	Mean left diaphragm thickness A+B (mm)
CT1	7.04+/-2.07	6.85+/-1.74	5.74+/-1.86	5.63+/-1.45	6.94+/-1.67	5.68+/-1.49
CT2	6.66+/-1.84	6.05+/-1.48	5.56+/-1.73	4.72+/-1.38	6.35+/-1.54	5.14+/-1.42

**Figure 3:**
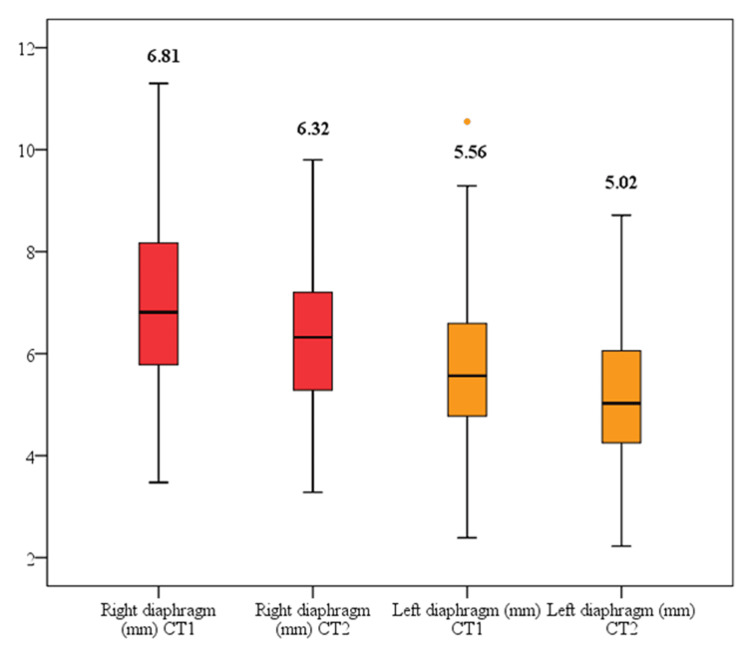
Diaphragm alteration during ICU hospitalization Box-plot comparing right and left diaphragm decrease in thickness among the enrolled patients at different CT scan investigation times. Red box plots represent right diaphragm measurements, whereas yellow box plots represent left diaphragm measurements. The horizontal lines within the boxes represent the median; the boxes represent the interquartile range; the ends of the whiskers represent the minimum and maximum values, excluding the outliners. The differences between the decreases in diaphragm thickness were assessed by applying a Student t-test.

Table 4 and Figure 3 present the dynamic diaphragm decrease in thickness for the entire cohort of included patients. The mean difference between CT (CT1 and CT2) scan investigations was -0.58 mm for the right diaphragm and -0.54 mm for the left diaphragm. Between the two CT scan investigations, the diaphragm thickness was significantly decreased on both the right and the left sides (p = 0.02).

**Table 4:** Mean dynamic diaphragm decrease in thickness for the whole cohort CT1 = first CT scan investigation; CT2 = second CT scan investigation; Student t-test.

Dynamic diaphragm decrease in thickness	Mean difference (mm)	P-value (CT1 and CT2)
Right diaphragm	-0.58	0.02
Left diaphragm	-0.54	0.02

Interreader variability assessment and correlation between diaphragm decrease in thickness 

Interreader variability (Table 5) was assessed by the Bland-Altman method. A total of 150 CT scans were assessed from 75 patients. Good A and B intervariability results were demonstrated: for the first CT scan investigation, the mean was 0.2 for the right diaphragm with an agreement interval of -3.5 to 3.9, as well as a mean of 0.1 for the left diaphragm with an agreement interval of -2.8 to 3.0; for the second CT scan investigation, a slight deviation from 0 was noticed, the mean was 0.6 for the right diaphragm, with an agreement interval of -1.9 to 3.2, and a mean of 0.8 for the left diaphragm with an agreement interval of -1.7 to 3.4.

**Table 5:** Inflammatory response WBC = white blood cells (normal range 3.6–10x10^3^/μL); Neutrophils (normal range 1.4–6.5x10^3^/μL); Lymphocytes (normal range 1.2–3.4x10^3^/μL); Platelets (normal range 150–450x10^3^/μL); NLR = neutrophil-lymphocyte ratio; PLR = platelets-lymphocyte ratio; SII = systemic inflammatory index = neutrophil count x monocyte/lymphocyte count; CRP = C-reactive protein (normal range 0–5 mg/L) §quantitative results available for 64 patients, qualitative (positive) results for 10 patients, 1 missing result; IL-6 = Interleukin-6 (normal range 0–7 pg/mL), §§25 missing results; * = Mann-Whitney U test.

	All patients (n=75)	Deceased (n=56)	Survivors (n=19)	p*	Right diaphragm with decrease in thickness	Right diaphragm without decrease in thickness	p*	Left diaphragm with decrease in thickness	Left diaphragm without decrease in thickness	p*
WBC (n=75) (min-max)	10.21 (2.27-56.5)	10.11 (2.27-56.55)	11.7 (5.72-41.53)	0.16	10.16 (2.27-56.55)	10.22 (3.44-29.0)	0.50	10.15 (2.27-56.5)	11.8 (4.82-37.4)	0.32
Neutrophils (n=75) (min-max)	8.84 (1.56-52.32)	8.66 (1.56-52.32)	10.61 (4.65-39.7)	0.18	8.57 (1.56-52.3)	9.11 (2.01-23.1)	0.50	8.38 (1.56-52.3)	10.4 (3.6-33.0)	0.29
Lymphocytes (n=75) (min-max)	0.78 (0.19-3.64)	0.77 (0.19-3.940)	0.85 (0.42-2.31)	0.13	0.79 (0.26-2.31)	0.67 (0.19-3.94)	0.77	0.79 (0.19-2.85)	0.74 (0.32-3.94)	0.63
Platelets (n=75) (min-max)	217.0 (35.0-1081.0)	204.50 (35.0-641.0)	298.5 (90.0-1081.0)	0.01	210.0 (38.2-1081.0)	230.0 (35.0-404.0)	0.95	207.0 (35.0-1081.0)	253.0 (127.0-420.0)	0.26
NLR (n=75) (min-max)	11.9 (1.48-106.77)	11.27 (1.48-106.77)	12.4 (3.75-52.28)	0.57	11.97 (1.48-106.7)	12.4 (2.08-66.63)	0.97	11.15 (1.48-106.7)	13.9 (2.08-10.04)	0.25
PLR (n=75) (min-max)	278.4 (52.2-1184.2)	273.63 (52.23-1174.21)	356.25 (105.8-1081.57)	0.29	276.3 (86.8-1081.57)	305.7 (52.2-1184.2)	0.57	274.6 (52.2-1184.2)	355.4 (59.6-773.8)	0.39
SII (n=75) (min-max)	2436.2 (262.9-42981.9)	2301.85 (262.9-24878.7)	3450.5 (952.1-42981.9)	0.10	2442.6 (262.9-42981.9)	2333.4 (482.1-14992.1)	0.83	2316.8 (262.9-42981.9)	3086.2 (612.6-13015.4)	0.21
CRP (n=64)^§^ (min-max)	91.36 (4.20-480.0)	102.68 (4.20-480.0)	70.5 (5.66-303.23)	0.25	90.9 (4.2-480.0)	117.1 (21.0-303.23)	0.98	88.2 (4.2-480.0)	92.5 (5.6-247.0)	0.80
IL -6 (n=50) ^§§^ (min-max)	50.1 (1.5-1768.0)	70.1 (1.50-1105.0)	26.38 (4.46-1738.0)	0.29	54.1 (1.5-1768.0)	45.5 (11.1-625.9)	0.61	70.1 (1.5-1768.0)	46.6 (10.4-184.1)	0.65

Inflammatory and metabolic response

Table 6 lists the inflammatory markers and parameters.

**Table 6:** Metabolic response Albumin (g/dL, <3.2 g/dL low serum level); Glucose (g/dL, ≥180 g/dL high serum glucose); Creatinine (mg/dL, normal range 0.7–1.20 mg/dL); GFR, glomerular filtration rate (mL/min, reference value >60 mL/min); Fibrinogen (mg/dL, normal range 150–400 mg/dL), §missing 26 laboratory values; Ferritin (ng/mL, normal range 15–150 ng/mL), §§missing 10 laboratory values; *Mann-Whitney U test; ** Student t-test.

Metabolic markers	All patients (n=75)	Deceased (n=56)	Survivors (n=19)	P	Right diaphragm with decrease in thickness	Right diaphragm without decrease in thickness	p	Left diaphragm with decrease in thickness	Left diaphragm without decrease in thickness	p
Albumin (n=64)	2.87+/-0.52	2.85+/-0.55	2.90+/-0.46	0.73**	2.82+/-0.50	3.04+/-0.58	0.16	2.89+/-0.54	2.81+/-0.46	0.61
Glucose (n=75)	176.7+/-94.7	179.4+/-89.2	168.7+/-111.7	0.67**	176.6+/-104.5	176.9+/-44.7	0.98	172.1+/-94.6	191.3+/-96.0	0.44
Creatinine (n=75)	2.55+/-1.5	3.05+/-1.98	1.019+/-0.39	0.44**	2.93+/-1.07	1.15+/-0.51	0.51	2.88+/-1.8	1.53+/-1.10	0.60
GFR (n=75)	75.6+/-72.8	73.7+/-81.2	81.3+/-40.0	0.69**	77.59+/-80.7	68.6+/-30.1	0.49	79.9+/-80.8	62.1+/-36.2	0.36
Fibrinogen (n=59)^ §^	446.0 (107.33-970.1)	441.7 (107.3-970.3)	486.7 (114.3-656.0)	0.74*	474.4 (114.3-970.0)	267.0 (107.3-775.7)	0.13	446.0 (108.8-970.1)	453 (107.3-868.9)	0.99
Ferritin (n=65)^ §§^	817.4 (59.9-35180.0)	817 (59.9-35180.0)	822.0 (77.7-3391.0)	0.87*	809.4 (59.9-35180.0)	960.7 (152.3-5403.0)	0.82	828.7 (59.9-35180.0)	728.7 (131.4-11257.5)	0.97

The laboratory results for all patients identified WBC values of 10.21 (2.27-56.5) 10^3^/μL, and they confirmed a clinical evolution of neutrophilia 8.84 (1.56-52.32) 10^3^/μL and lymphopenia 0.78 (0.19-3.64) 10^3^/μL, with increased CRP values of 91.36 (4.20-480.0) mg/L and increased IL-6 values of 50.1 (1.5-1,768.0). Increased absolute neutrophil count and decreased absolute lymphocyte count contributed to an elevated NLR = 11.9 (1.48-106.77), which indicated at least a moderate physiologic stress level. The other indicators of inflammation PLR = 278.4 (52.2-1184.2) and SII = 2,436.2 (262.9-42,981.9) showed an imbalance in the critically ill patients’ inflammatory status. There were no significant differences between survivors and deceased regarding WBC (p = 0.16), neutrophils (p = 0.18), lymphocytes (p = 0.13), NLR (p = 0.57), PLR (p = 0.29), SII (p = 0.10), CRP (p = 0.25), and IL-6 (p = 0.29). The same statistically nonsignificant results are obtained when comparing the group with the decrease in diaphragm thickness versus the group without a decrease in diaphragm thickness, both for the right and left sides, regarding all the inflammatory markers and parameters (p > 0.05).

No statistically significant differences were obtained when inflammatory markers such as albumin, fibrinogen, and ferritin were compared in the patient groups. Table 7 presents all the results in this respect.

**Table 7:** Correlation between diaphragm thickness and LOS in ICU and LOS in hospital LOS ICU = length of stay in intensive care unit; LOS Hospital = length of stay in hospital; Delta right diaphragm (mm) = the difference between mean values of the right diaphragm obtained in the first and second CT scan by A and B investigators; Delta left diaphragm (mm) = the difference between mean values of the left diaphragm obtained in the first and second CT scan by A and B investigators; Spearman rho correlation coefficient was calculated.

	Delta right diaphragm thickness		Delta left diaphragm thickness (mm)	
	Correlation coefficient (rho)	p-value	Correlation coefficient (rho)	p-value
LOS ICU (days)	-0.056	0.635	-0.149	0.201
LOS Hospital (days)	-0.174	0.135	-0.227	0.050

Diaphragm alterations over ICU hospitalization

To assess a possible linear association between a decrease in diaphragm thickness and length of stay in the ICU and between the decrease in diaphragm thickness and length of stay in the hospital, a correlation coefficient was determined (Table 8).

**Table 8:** AUC and cut-off values for the diaphragm alterations, mechanical ventilation, and ICU mortality MV = mechanical ventilation; AUC values were displayed including 95% CI and compared according to Hanley and McNeil’s method.

	AUC	The lower bound (95% CI)	The upper bound (95% CI)	Cut-off value (mm)
Right diaphragm				
≤24 hr MV	0.552	0.433	0.667	-0.535
25–96 hr MV	0.614	0.494	0.724	-0.355
>96 hr MV	0.516	0.398	0.633	-0.125
ICU mortality	0.553	0.434	0.668	-0.175
Left diaphragm				
≤24 hr MV	0.581	0.462	0.694	-0.055
25–96 hr MV	0.614	0.494	0.724	-0.355
>96 hr MV	0.548	0.428	0.663	-0.055
ICU mortality	0.606	0.487	0.717	-0.435

Negative coefficient correlation for the right diaphragm (-0.056 regarding LOS in ICU; -0.174 regarding LOS Hospital) and the left diaphragm (-0.149 regarding LOS in ICU; -0.227 regarding LOS Hospital) demonstrates the duration of hospitalization prolongations, and the diaphragm decreases in thickness, but the results are not statistically significant (p > 0.05) for both the right and left diaphragm.

ROC curves assessing the decrease in diaphragm thickness for prognosis and MV

**Figure 4:**
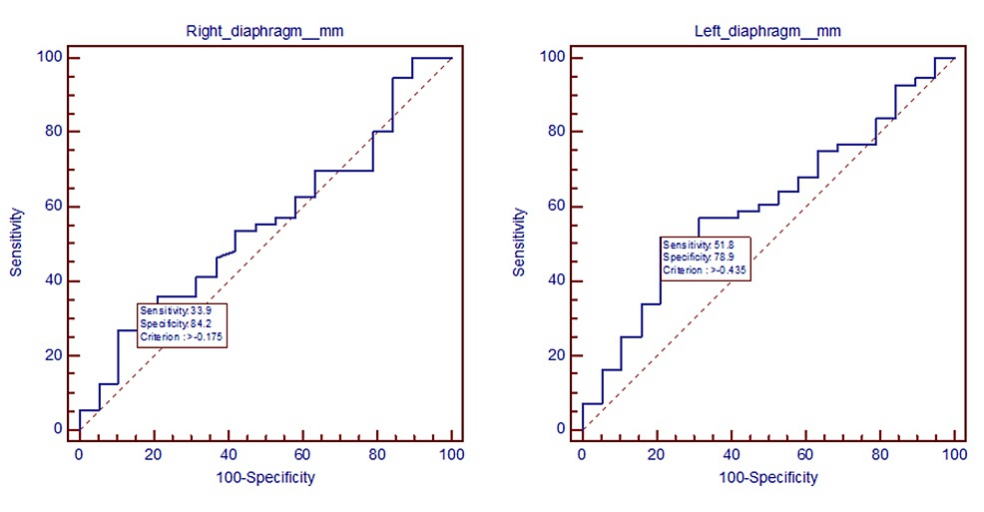
ROC-AUC for diaphragm thickness and patients' prognosis The figure represents the graphical plot used to show the right diaphragm criterion for poor prognosis (>-0.175), with a sensitivity of 33.9% and a specificity of 84.2% and the left diaphragm criterion for poor prognosis (>-0.435), with a sensitivity of 51.8% and a specificity of 78.9%.

ROC curve assessed diaphragm thickness and prognosis. Survival curves were plotted for each side of the diaphragm (Figure 4).

**Figure 5:**
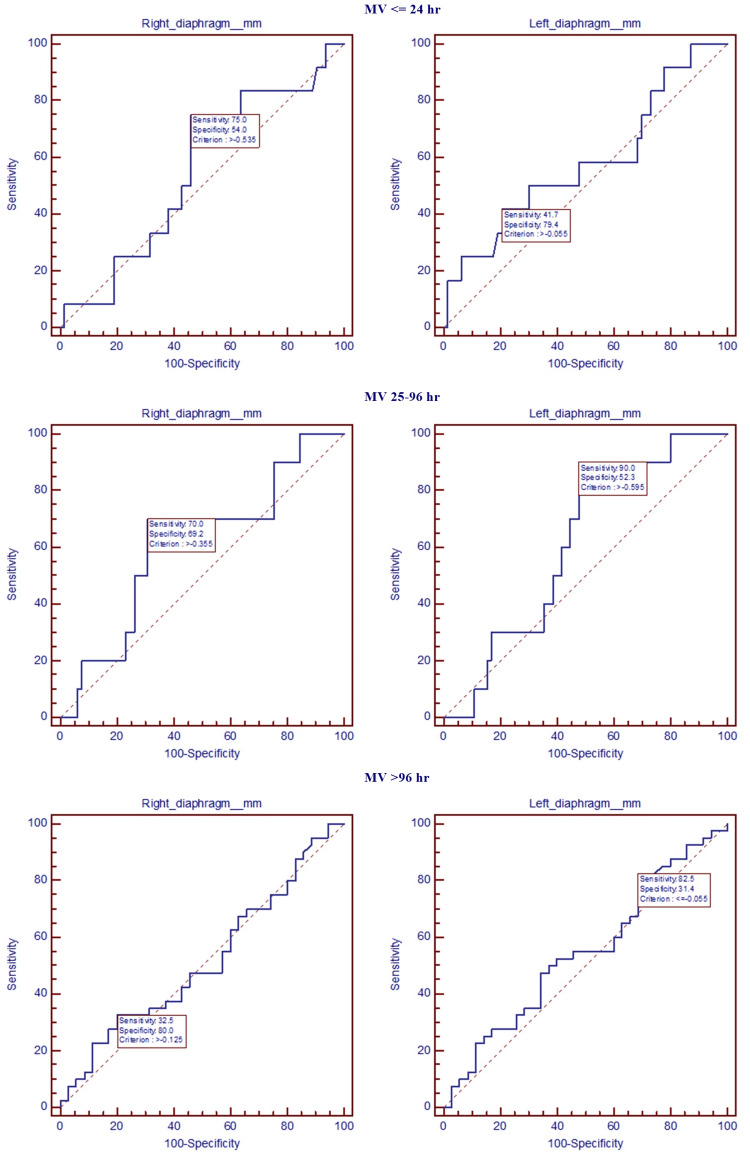
ROC curves for diaphragm alterations and mechanical ventilation Prediction for right diaphragm changes regarding the period of MV (≤24 hr: criterion >-0.535, sensitivity 75%, specificity 54%; 25–96 hr: criterion >-0.355, sensitivity 70.0%, specificity 69.2%; >96 hr: criterion >-0.125, sensitivity 32.5%, specificity 80.0%) and prediction for left diaphragm changes regarding the period of MV (≤24 hr: criterion>-0.055, sensitivity 41.7%, specificity 79.4%; 25–96 hr: criterion >-0.595, sensitivity 90.0%, specificity 52.3%; >96 hr: criterion ≤-0.055, sensitivity 82.5%, specificity 31.4%).

The ROC curves corresponding to alterations in diaphragm structure and the application of MV, serve to illustrate the underlying tradeoff between sensitivity and specificity (Figure 5). All curves were plotted for each side of the diaphragm.

The AUC of the decrease in diaphragm thickness for evaluating the risk of prolonged mechanical (>96 hr) ventilation and ICU mortality are analyzed in Table 9. The cut-off values indicate a high risk of prolonged MV (-0.125 for the right diaphragm and -0.055 for the left diaphragm) and a high risk of ICU mortality (-0.175 for the right diaphragm and -0.435 for the left diaphragm).

**Table 9:** Logistic regression for left and right diaphragm, MV, LOS, and outcome in critically ill patients MV = mechanical ventilation; LOS ICU = length of stay in intensive care unit; LOS Hospital = length of stay in hospital; right and left diaphragm: binary and dependent variables; MV, LOS, and outcome: independent variables; OR = odds ratio; CI = confidence interval.

Right diaphragm	P-value	OR	Lower 95% CI for OR	Upper 95% CI for OR
MV ≤24 hr	0.579	0.383	0.013	11.365
MV 25–96 hr	0.626	0.440	0.016	11.983
MV >96 hr	0.383	0.348	0.001	5.679
LOS ICU (days)	0.769	1.006	0.966	1.047
LOS hospital (days)	0.241	1.004	0.972	1.121
Outcome hospital	0.853	1.263	0.106	15.081
Left diaphragm	P-value	OR	Lower 95% CI for OR	Upper 95% CI for OR
MV ≤24 hr	0.127	0.364	0.09	0.127
MV 25–96 hr	0.634	0.700	0.161	0.634
MV >96 hr	0.163	2.160	0.731	0.163
LOS ICU (days)	0.597	1.012	0.968	0.597
LOS hospital (days)	0.590	1.008	0.979	0.590
Outcome hospital	0.316	0.512	0.130	0.316

Logistic regression with respect to MV, length of stay, and outcome

Demonstrating logistic regression, this study reveals the ORs for both the right and the left diaphragm are greater than 1 regarding LOS and outcome. For the right diaphragm, it is observed LOS ICU, OR = 1.006 (95% CI = 0.966-1.047); LOS Hospital, OR = 1.004 (95% CI = 0.972-1.121); Outcome hospital, OR = 1.263 (95% CI = 0.106-15.081). For the left diaphragm, it is observed LOS ICU, OR = 1.012 (95% CI = 0.968-0.597); LOS Hospital, OR = 1.008 (95% CI = 0.979-0.590). These results indicate the event is more likely to occur as the predictor increases. The MV ORs were less than 1, indicating the event is less likely to occur as the predictor increases. The 95% confidence level indicates the CI contains the OR value for the population. Still, as the interval is too wide, the sample size should be increased.

## Discussion

Being the main respiratory muscle, the diaphragm is impaired upon increased breathing efforts [13,22]. Concerning the alteration of the muscle, SARS-CoV-2 infection looks to be equal to other causes of diaphragmatic dysfunction (central nervous system or peripheral neuron disorders, neuromuscular junction disorders, contractile diaphragm disorders) [13,23,24]. As the immediate diagnosis of diaphragmatic dysfunction in critically ill patients is of great importance for therapeutic management, this study assessed positive COVID-19 patients’ diaphragm changes. The main findings observed in this study are thus as follows.

(1) The mean difference in the decrease of diaphragm muscle thickness for patients admitted to the ICU for at least 24 hr and having two diaphragm CT scan investigations was -0.58 mm for the right diaphragm and -0.54 mm for the left diaphragm, which is statistically significant (p = 0.02).

(2) The decrease in diaphragm thickness was associated negatively, but not statistically significant, with LOS ICU and LOS in hospital. Additionally, no statistically significant differences were noticed between the group with diaphragm thickness decrease and the group without diaphragm thickness decrease regarding inflammatory and metabolic responses (p > 0.05).

(3) AUC and cut-off values for high risk of prolonged MV and ICU mortality were analyzed, respectively, for the OR for MV, LOS in ICU, LOS in hospital, and the outcome.

On the one hand, in 2016, Lee et al. presented the results of 13 mechanically ventilated patients for whom measurement of diaphragm thickness on chest CT was determined [21]. Comparing the two studies, similar aspects regarding demographics (age, sex distribution) and the cause of ICU admission (the majority hospitalized for ARF) can be seen. Lee concluded the thickness of the diaphragm was significantly decreased on both the left and right sides (p < 0.01) [21]. Evaluating the results of our study, a statistically significant difference in diaphragm decrease can be seen for the right side (p = 0.02, mean difference = -0.58 mm) and the left side (p = 0.02, mean difference = -0.54 mm). On the other hand, Parlak et al. published a retrospective analysis of 404 positive COVID-19 patients admitted to the ICU and non-ICU departments [12]. Their results referred to bilateral diaphragm thickness measurement, presenting a statistically significant relationship between the presence of a thinner diaphragm and mortality (p < 0.001) [12]. Negative correlation coefficients were obtained for right diaphragm thickness and LOS in ICU (-0.056) and LOS in hospital (-0.174), for the left diaphragm thickness and LOS in ICU (-0.149) and LOS in hospital (-0.227), but they were not statistically significant (p = 0.635; p = 0.135; p = 0.201; p = 0.050).

In addition, exploring the inflammatory response, it has been remarked patients are characterized by neutrophilia (8.84 (1.56-52.32) 10^3^/μL), lymphopenia (0.78 (0.19-3.64) 10^3^/μL), high results of NLR (11.9 (1.48-106.77)), PLR (278.4 (52.2-1,184.2)), SII (2436.2 (262.9-42,981.9)) and high values of CRP = 91.36 (4.20-480) mg/L, IL-6 = 50.1 (1.5-1,768.0) pg/mL. Used as tools and markers of COVID-19 disease severity and prognosis, these parameters were investigated by Moisa et al. to describe their predictive value for MV and death [25]. Even if dynamic changes in hematological indices were strongly correlated with disease progression and severity [25], the results of our study found no statistically significant differences between deceased and survivors between patients with and without a decrease in diaphragm thickness (p > 0.05).

In our study, the metabolic response results were reflected by hypoalbuminemia (albumin = 2.87+/-0.52 g/dL), which was used as an indicator of malnutrition, kidney injury (increased creatinine = 2.55+/-1.5 mg/dL), hyperglycemia (glucose = 176.7+/-94.7 g/dL), hyperfibrinogenemia (fibrinogen = 446.0 (107.33-970.1) mg/dL), and hyperferritinemia (ferritin = 817.4 (59.9-3,5180.0) ng/mL). The metabolic laboratory results are not statistically significant between survivors and the deceased between the group with and without diaphragm decrease in thickness (p > 0.05). Thus, further and more extensive studies are needed to evaluate the exact impact of systemic inflammation upon diaphragm modification in mechanically ventilated patients.

Compared to Parlak’s interobserver agreement for measurements, where almost a perfect agreement was found (interclass correlation coefficient of 0.904 (95% CI 0.759-0.954) [12], in this study a good agreement for diaphragm thickness measurement was obtained in interreader variability assessment (see Table 5; CT1: concordance correlation coefficient of 0.518 (95% CI 0.337-0.662) for the right diaphragm and of 0.609 (95% CI 0.454-0.728) for the left diaphragm. Moreover, in CT2, the concordance correlation coefficient of 0.654 (95% CI 0.518-0.757) for the right diaphragm and 0.568 (95% CI 0.423-0.686) for the left diaphragm was noted).

Measurement of muscle wasting was found to add data regarding the evolution of patients’ diseases, either assessed by ultrasound in the ICU [26] or by CT investigations in critical and noncritical settings [27,28]. Even if various studies evaluated patients diagnosed with common respiratory disorders and their associated risks [29], with reference to COVID-19 patients, Parlak et al. evaluated the association of diaphragm thickness and density measured with chest CT with disease severity [12], and Branea et al. investigated diaphragm alterations on CTs of critically ill patients [30]. Related to these findings, our study appreciated the AUC and cut-off values for diaphragm decrease in thickness and prolonged MV (>96 hr): AUC = 0.516 (95% CI = 0.398-0.633) for the right diaphragm, AUC = 0.548 (95% CI = 0.428-0.663) for the left diaphragm; and ICU mortality: AUC = 0.553 (95% CI = 0.434-0.668) for the right diaphragm, AUC = 0.606 (95% CI = 0.487-0.717) for the left diaphragm. A similar ROC analysis of the total mean diaphragm thickness for the prediction of severe disease and mortality was found in Parlak’s study: AUC = 0734 (95% CI = 0.669-0.800) and AUC = 0.717 (95% CI = 0.607-0.828) [12]. Our study analyzed the OR for diaphragm decrease in thickness and LOS in ICU, LOS in hospital, and hospital outcome. 

Although the literature on this subject is scarce, because it is relatively easy to evaluate it on a CT scan, a diaphragm thickness assessment may be useful in positive COVID-19 cases and can be extended to the mechanically ventilated critically ill. This need can be fulfilled by enrolling more patients, the reduced number of patients evaluated being a limit of this study when it comes to expressing the exact recommendations for diaphragm measurements in mechanically ventilated patients.

## Conclusions

CT diaphragm evaluation in COVID-19 mechanically ventilated patients has the possibility of becoming a reliable tool in predicting muscle modifications. Further studies with regard to the ventilation management of these patients, based on their diaphragm alterations, are encouraged, with the purpose of achieving a personalized treatment for mechanically ventilated patients.

## References

[1] El Zowalaty ME, Järhult JD (2020). From SARS to COVID-19: a previously unknown SARS- related coronavirus (SARS-CoV-2) of pandemic potential infecting humans - call for a One Health approach. One Health.

[2] Hu B, Guo H, Zhou P, Shi ZL (2021). Characteristics of SARS-CoV-2 and COVID-19. Nat Rev Microbiol.

[3] Msemburi W, Karlinsky A, Knutson V, Aleshin-Guendel S, Chatterji S, Wakefield J (2023). The WHO estimates of excess mortality associated with the COVID-19 pandemic. Nature.

[4] Sohrabi C, Alsafi Z, O'Neill N (2020). World Health Organization declares global emergency: a review of the 2019 novel coronavirus (COVID-19). Int J Surg.

[5] Nicola M, O'Neill N, Sohrabi C, Khan M, Agha M, Agha R (2020). Evidence based management guideline for the COVID-19 pandemic - review article. Int J Surg.

[6] Li X, Ma X (2020). Acute respiratory failure in COVID-19: is it "typical" ARDS?. Crit Care.

[7] Mutair Al A, Alhumaid S, Layqah L (2022). Clinical outcomes and severity of acute respiratory distress syndrome in 1154 COVID-19 patients: an experience multicenter retrospective cohort study. COVID.

[8] Rana A, Singh H, Mavuduru R, Pattanaik S, Rana PS (2022). Quantifying prognosis severity of COVID-19 patients from deep learning based analysis of CT chest images. Multimed Tools Appl.

[9] Yu Z, Li X, Sun H (2020). Rapid identification of COVID-19 severity in CT scans through classification of deep features. Biomed Eng Online.

[10] Yoon JK, Lee S, Kim KW, Lee JE, Hwang JA, Park T, Lee J (2021). Reference values for skeletal muscle mass at the third lumbar vertebral level measured by computed tomography in a healthy Korean population. Endocrinol Metab (Seoul).

[11] You Y, Chen M, Chen X, Yu W (2022). Diaphragm thickness on computed tomography for nutritional assessment and hospital stay prediction in critical COVID-19. Asia Pac J Clin Nutr.

[12] Parlak S, Beşler MS, Gökhan MB (2022). Association of diaphragm thickness and density measured on chest CT with disease severity in COVID-19 patients. Am J Emerg Med.

[13] Laghi FA Jr, Saad M, Shaikh H (2021). Ultrasound and non-ultrasound imaging techniques in the assessment of diaphragmatic dysfunction. BMC Pulm Med.

[14] Dres M, Goligher EC, Heunks LM, Brochard LJ (2017). Critical illness-associated diaphragm weakness. Intensive Care Med.

[15] Lad H, Saumur TM, Herridge MS, Dos Santos CC, Mathur S, Batt J, Gilbert PM (2020). Intensive care unit-acquired weakness: not just another muscle atrophying condition. Int J Mol Sci.

[16] Shanely RA, Van Gammeren D, Deruisseau KC, Zergeroglu AM, McKenzie MJ, Yarasheski KE, Powers SK (2004). Mechanical ventilation depresses protein synthesis in the rat diaphragm. Am J Respir Crit Care Med.

[17] Levine S, Nguyen T, Taylor N (2008). Rapid disuse atrophy of diaphragm fibers in mechanically ventilated humans. N Engl J Med.

[18] Charlson ME, Pompei P, Ales KL, MacKenzie CR (1987). A new method of classifying prognostic comorbidity in longitudinal studies: development and validation. J Chronic Dis.

[19] Haimovich AD, Ravindra NG, Stoytchev S (2020). Development and validation of the quick Covid-19 severity index: a prognostic tool for early clinical decompensation. Ann Emerg Med.

[20] Levine DM, Lipsitz SR, Co Z, Song W, Dykes PC, Samal L (2021). Derivation of a clinical risk score to predict 14-day occurrence of hypoxia, ICU admission, and death among patients with coronavirus disease 2019. J Gen Intern Med.

[21] Lee GD, Kim HC, Yoo JW, Lee SJ, Cho YJ, Bae K, Lee JD (2016). Computed tomography confirms a reduction in diaphragm thickness in mechanically ventilated patients. J Crit Care.

[22] Laghi F, Tobin MJ (2003). Disorders of the respiratory muscles. Am J Respir Crit Care Med.

[23] McCool FD, Manzoor K, Minami T (2018). Disorders of the diaphragm. Clin Chest Med.

[24] Dubé BP, Dres M (2016). Diaphragm dysfunction: diagnostic approaches and management strategies. J Clin Med.

[25] Moisa E, Corneci D, Negoita S, Filimon CR, Serbu A, Negutu MI, Grintescu IM (2021). Dynamic changes of the neutrophil-to-lymphocyte ratio, systemic inflammation index, and derived neutrophil-to-lymphocyte ratio independently predict invasive mechanical ventilation need and death in critically ill COVID-19 patients. Biomedicines.

[26] Branea OE, Jugariu AR, Budeanu RG, Copotoiu SM, Copotoiu M (2018). Ultrasonography: New insights in its applicability to explore muscle mass and musculoskeletal inflammation in critically ill patients. Acta Medica Marisiensis.

[27] Bak SH, Kwon SO, Han SS, Kim WJ (2019). Computed tomography-derived area and density of pectoralis muscle associated disease severity and longitudinal changes in chronic obstructive pulmonary disease: a case control study. Respir Res.

[28] Park MJ, Cho JM, Jeon KN (2014). Mass and fat infiltration of intercostal muscles measured by CT histogram analysis and their correlations with COPD severity. Acad Radiol.

[29] Ardelean CL, Pescariu S, Lighezan DF, Pleava R, Ursoniu S, Nadasan V, Mihaicuta S (2019). Particularities of older patients with obstructive sleep apnea and heart failure with mid-range ejection fraction. Medicina (Kaunas).

[30] Branea OE, Budeanu AR, Budeanu RG, Chiuzan AȘ, Nazaret IL, Copotoiu SM, Lazăr AE (2022). Computed tomography evaluation of diaphragm alterations in 20 critically ill patients. Acta Medica Marisiensis.

